# Association of *TGF-β1* Polymorphisms with Breast Cancer Risk: A Meta-Analysis of Case–Control Studies †

**DOI:** 10.3390/cancers12020471

**Published:** 2020-02-18

**Authors:** B. Madhu Krishna, Samir Jana, Aditya K. Panda, David Horne, Sanjay Awasthi, Ravi Salgia, Sharad S Singhal

**Affiliations:** 1 Department of Medical Oncology, Beckman Research Institute of City of Hope, Comprehensive Cancer Center and National Medical Center, Duarte, CA 91010, USAsajana@coh.org (S.J.); rsalgia@coh.org (R.S.); 2 Department of Bioscience and Bioinformatics, Khallikote University, Konisi, Berhampur, Odisha 761008, India; adityarmrc@gmail.com; 3 Department of Molecular Medicine, Beckman Research Institute of City of Hope, Comprehensive Cancer Center and National Medical Center, Duarte, CA 91010, USA; dhorne@coh.org; 4 Department of Internal Medicine, Division of Hematology & Oncology, Texas Tech University Health Sciences Center, Lubbock, TX 79430, USA; sanjay.awasthi@ttuhsc.edu

**Keywords:** breast cancer, polymorphism, TGFβ, meta-analysis

## Abstract

Reports on the association of *TGF-β1* polymorphisms with breast cancer (BC) have been conflicting, inconsistent, inconclusive, and controversial. PubMed, EMBASE, and Google Scholar were used to identify studies on *TGF-β1* polymorphisms and BC risk. Data were extracted independently, and of the initial 3043 studies, 39 case-control studies were eligible for inclusion in the meta-analysis. Information from these studies was extracted, and the overall associations of three *TGF-β1* polymorphisms (*TGF-β1* 29>T/C, *TGF-β1*-509 C/T, and TGF-β1*6A) with BC risk were analyzed using overall allele, homozygous, heterozygous, recessive, and dominant models. None of the three *TGF-β1* polymorphisms studied had a significant influence on the development of BC. However, stratified analysis revealed a positive correlation between the *TGF-β1* 29T>C polymorphism and BC risk according to a heterozygous model of the Asian population (odds ratio (OR) = 1.115, 95% confidence interval (CI) = 1.006–1.237, *p* = 0.039). Interestingly, this polymorphism was associated with lower odds of BC according to a heterozygous model of the Middle Eastern population (OR = 0.602, 95% CI = 0.375–0.966, *p* = 0.035). Thus, our analysis of large datasets indicates that the *TGF-β1* 29T>C polymorphism is significantly associated with BC risk in the Asian population. In contrast, the TGF-β1*6A and *TGF-β1-509 C/T* polymorphisms failed to show an association with BC.

## 1. Introduction

The transcription growth factor β (TGF-β) signaling pathway has been studied extensively in several cancer types, including breast cancer (BC). The TGF-β superfamily consists of 33 structurally similar members, including bone morphogenic proteins (BMPs), TGF-β ligands, and activins, which play important roles in mammary development [1,2,3]. In humans, isoforms of TGF-β ligands that are closely related both structurally and functionally (TGF-β1, TGF-β2, and TGF-β3) have been reported to modulate cell progression, migration, and apoptosis [4]. During cancer development, TGF-β exhibits both tumorigenic and tumor-suppressive roles [5]. It has been reported that, whereas TGF-β acts as a tumor suppressor during the early stages of cancer development, it exhibits oncogenic properties, inducing cell migration and invasion, during the later stages [2,6,7].

Several polymorphisms have been identified in the *TGF-β1* gene (*TGFβ1*), including two located in exon 1 (*TGF-β1* 29T>C and TGF-β1*6A) and two located in the promoter region (*TGF-β1*-509 C/T and *TGF-β1*-800 G/A) [8,9,10]. There is increasing evidence of an association between these polymorphisms and elevated cancer risk [11]. Researchers demonstrated that the *TGF-β1* 29T>C polymorphism increases the secretion of TGF-β levels [9]. Ziv et al. demonstrated that CC genotype of the *TGF-β1* 29T>C polymorphism decreases BC risk by up to 64% among Whites [12]. In contrast, Dunning et al. found that this polymorphism was associated with an increased risk of BC [9]. In a multi-ethnic cohort study, there was no association between this polymorphism and BC risk in postmenopausal Caucasian women [13]. However, a meta-analysis of case–control studies established a moderate association between the *TGF-β1* 29T>C polymorphism and BC susceptibility [14], and it included studies that either did not assess BC risk, lacked information regarding control samples, or did not incorporate data from more recent studies that might influence the combined ORs. Our present meta-analysis rectifies these previous mistakes by excluding studies that did not assess BC risk and/or did not have information regarding control samples.

The *TGF-β1*-509 C/T polymorphism, which is present in the promoter region of *TGF-β1,* influences its expression [15,16]. Elevated levels of *TGF-β1* in plasma were associated with the T allele of *TGF-β1*-509 C/T [16]. Several studies have assessed the association between *TGF-β1*-509 C/T polymorphism and BC risk [17]. This polymorphism has also been associated with radiation-induced fibrosis; however, contrasting results have also been recently obtained [18]. 

The deletion of three alanine residues within the nine-alanine repeat sequence (* 9A) of the *TGF*-*β* exon 1 region results in the formation of the TGF-β1*6A variant [10]. Studies have suggested that the TGF-β1*6A variant exhibits significantly fewer tumor-suppressive properties than wild-type *TGF-β1* [19]. TGF-β1*6A is a tumor-susceptibility allele that is highly correlated with the risk of various cancer types [20]. Baxter et al. found that the TGF-β1*6A variant increased BC risk by 60% [21]. However, contradictory results have also been observed [19,22,23,24,25]. Therefore, we performed the current meta-analysis to provide conclusive evidence for the associations between *TGF-β1* polymorphisms and BC risk.

## 2. Results

### 2.1. Characteristics of Studies Included in the Meta-Analysis

Initially, 3043 hits were obtained from publications on PubMed, EMBASE, and Google Scholar. Among them, 373 publications were reviews, which were excluded. The remaining studies were retrieved, scrutinized, and evaluated by reading their titles and abstracts. Eighty potentially eligible studies were retrieved as full texts, 39 of which were eligible for inclusion in the meta-analysis. Precisely, 23 studies [9,12,13,26,27,28,29,30,31,32,33,34,35,36,37,38,39,40,41,42,43,44,45] covered the *TGF-β1* 29T>C polymorphism (also known as Pro10Leu, T869C, rs1982073, and rs1800470), whereas 13 studies [9,28,29,30,35,40,46,47,48,49,50,51,52] covered the *TGF-β1*-509 C/T polymorphism (also known as −1349C>T and rs1800469), and 13 studies [9,21,28,31,33,35,39,47,53,54,55,56,57] covered the TGF-β1*6A polymorphism. Genotypic and allelic frequencies from each eligible case-control study were extracted. The characteristics of all eligible studies are represented in Table 1. Studies were sub-categorized based on the ethnicities of the individuals studied (Caucasian, European, Middle Eastern, and Asian), and were analyzed separately. Supplementary Figure S1 depicts the selection of studies in a comprehensive PRISMA flow chart.

### 2.2. Assessment of Heterogeneity among the Studies and Publication Bias

Heterogeneity was observed in the allelic models of all three *TGF-β1* polymorphisms, as well as all genetic models of the *TGF-β1* 29T>C polymorphism and the homozygous model of the *TGF-β1*-509 C/T polymorphism. In contrast, none of the genetic models of the TGF-β1*6A polymorphism were heterogeneous. Heterogeneity among the studies of all three polymorphisms in all ethnic groups is represented in Supplementary Table S1. Information regarding the presence of potential publication bias in ethnic studies is illustrated in Supplementary Figure S2.

### 2.3. TGF-β1 29T>C, or T869C, Polymorphism

**Figure 1:**
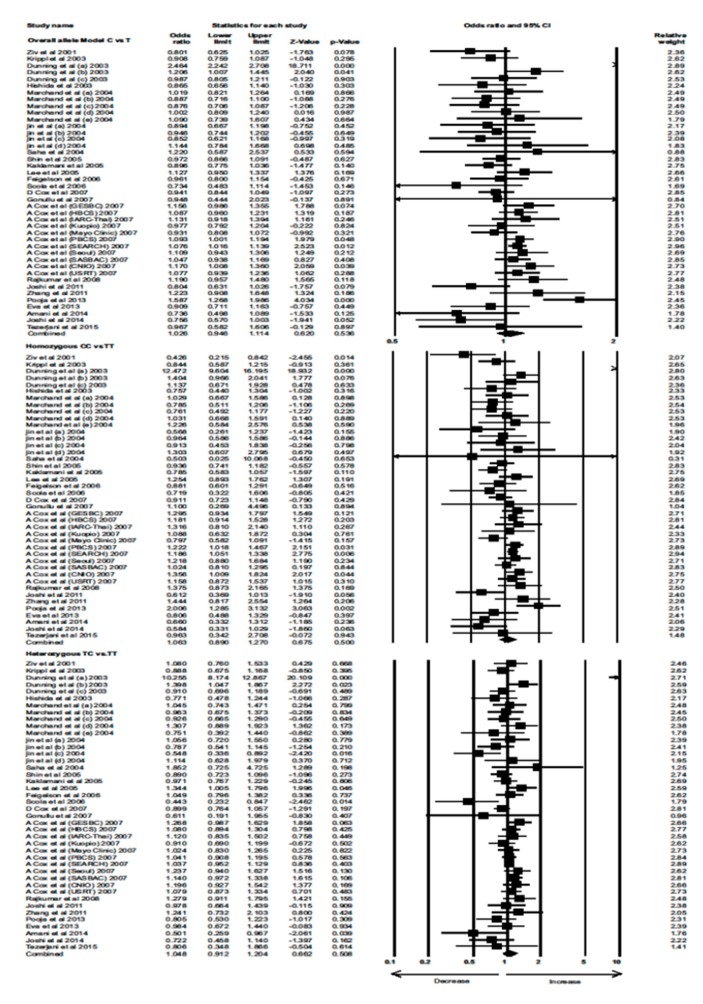
Forest plot of overall allele, homozygous, and heterozygous genotypic analyses of the *TGF-β1* 29T>C polymorphism and assessment of its association with BC risk using combined odds ratios (ORs) and 95% confidence intervals (CIs) of the ORs. Black squares represent the ORs of the individual studies, and horizontal lines indicate the 95% CIs of the ORs.

**Figure 2:**
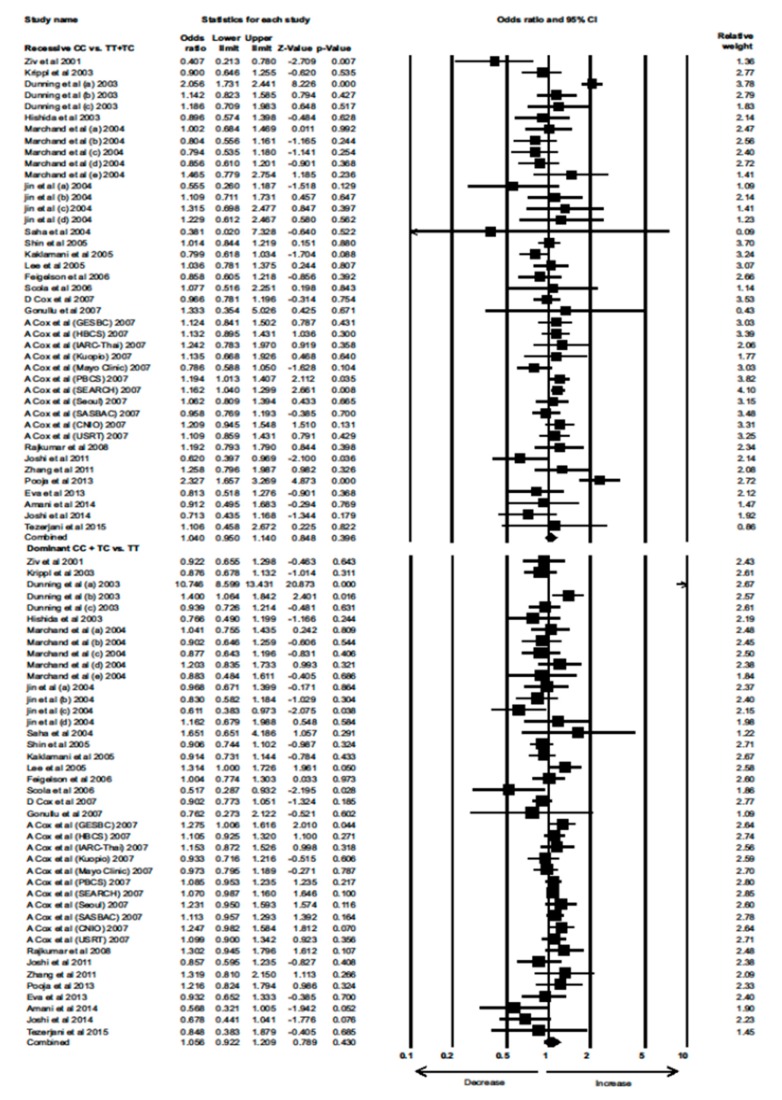
Forest plot of recessive and dominant genotypic analyses of the *TGF-β1* 29T>C polymorphism and assessment of its association with BC risk using combined odds ratios (ORs) and 95% confidence intervals (CIs) of the ORs. Black squares represent the ORs of the individual studies, and horizontal lines indicate the 95% CIs of the ORs.

Data from 23 eligible case–control studies were extracted to evaluate the association between the *TGF-β1* 29T>C polymorphism and BC risk. Interestingly, neither the overall allele model nor any of the genetic models showed a significant association between the *TGF-β1* 29T>C polymorphism with increased risk of BC (Combined analysis: overall allele (OR) = 1.026, 95% confidence interval (CI) = 0.946–1.114, *p* = 0.536; homozygous: OR = 1.063, 95% CI = 0.890–1.270, *p* = 0.500; heterozygous: OR = 1.048, 95% CI = 0.912–1.204, *p* = 0.508; recessive: OR = 1.040, 95% CI = 0.950–1.140, *p* = 0.396; dominant: OR = 1.056, 95% CI = 0.922–1.209, *p* = 0.430) (Figure 1 and Figure 2).

### 2.4. TGF-β1-509 C/T, or -1349C>T, Polymorphism 

**Figure 3:**
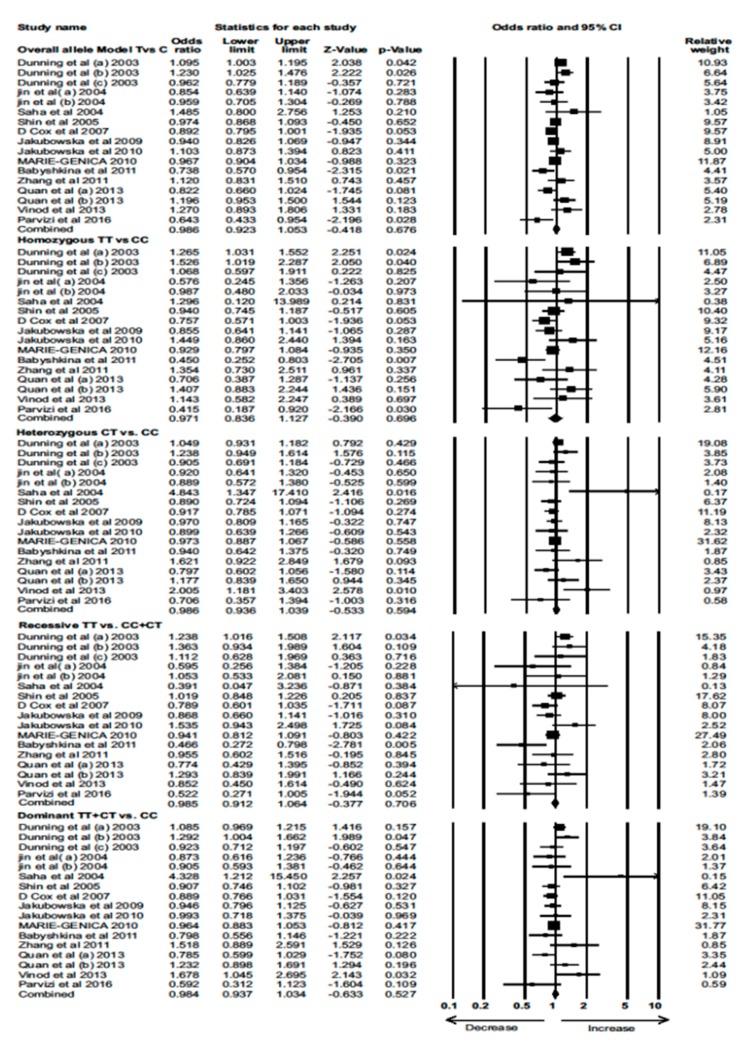
Forest plot of overall allele and genotypic analysis of the *TGF-β1*-509 C/T polymorphism and assessment of its association with BC risk using combined odds ratios (ORs) and 95% confidence intervals (CIs) of the ORs. Black squares represent the ORs of the individual studies, and horizontal lines indicate the 95% CIs of the ORs.

Data from 13 eligible case–control studies were extracted to evaluate the association between the *TGF-β1*-509 C/T polymorphism and BC risk. None of the models showed a significant association between the *TGF-β1*-509 C/T polymorphism and BC risk (Combined analysis: overall allele (OR)  =  0.986, 95% CI = 0.923–1.053, *p* = 0.676; homozygous: OR = 0.971, 95% CI = 0.836–1.127, *p* = 0.696; heterozygous: OR = 0.986, 95% CI = 0.936–1.039, *p* = 0.594; recessive: OR = 0.985, 95% CI = 0.912–1.064, *p* = 0.706; dominant: OR = 0.984, 95% CI = 0.937–1.034, *p* = 0.527) (Figure 3). 

### 2.5. TGF-β1*6A polymorphism

**Figure 4:**
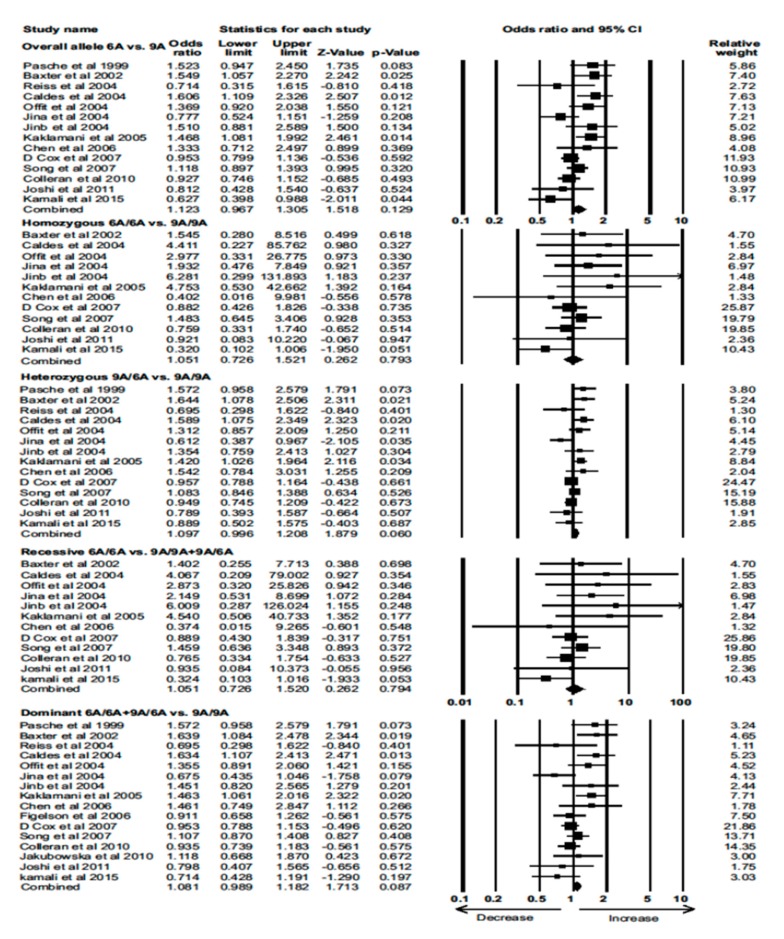
Forest plot of overall allele and genotypic analyses of the TGF-β1*6A polymorphism and assessment of its association with BC risk using pooled combined odds ratios (ORs) and 95% confidence intervals (CIs) of the ORs. Black squares represent the ORs of the individual studies, and horizontal lines indicate the 95% CIs of the ORs.

Data from 13 eligible case–control studies were extracted to evaluate the association between the TGF-β1*6A polymorphism and BC risk. Again, none of the models showed a significant association between the TGF-β1*6A polymorphism and BC (Combined analysis: overall allele (OR)  =  1.123, 95% CI = 0.967–1.305, *p* = 0.129; homozygous: OR = 1.051, 95% CI = 0.726–1.521, *p* = 0.793; heterozygous: OR = 1.097, 95% CI = 0.996–1.208, *p* = 0.060; recessive: OR = 1.051, 95% CI = 0.726–1.520, *p* = 0.794; dominant: OR = 1.081, 95% CI = 0.989–1.182, *p* = 0.087) (Figure 4). These results for each polymorphism did not differ by genotyping method (Supplementary Figures S3 and S4). 

### 2.6. Stratified Analysis 

**Figure 5:**
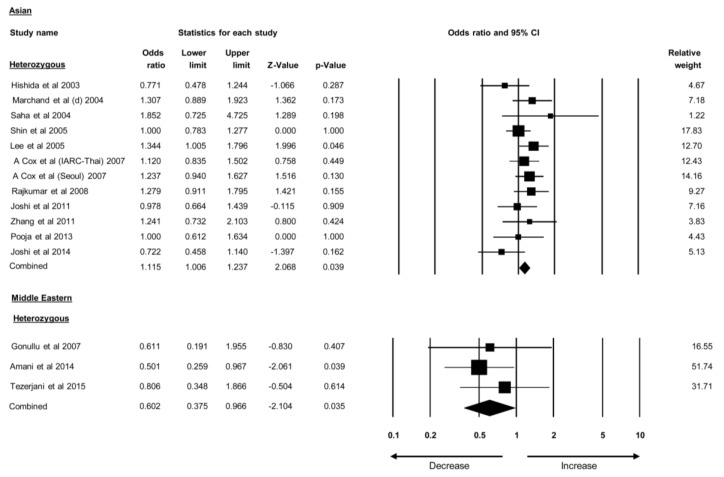
Forest plot of stratified analysis of *TGF-β1* polymorphisms that show association with either BC risk or lower odds in various ethnic groups. Black squares represent the odds ratios (ORs) of the individual studies, and horizontal lines indicate the 95% confidence intervals (CIs) of the ORs.

Studies were categorized based on the ethnicities of the individuals studied. Studies covering the *TGF-β1* 29T>C polymorphisms were divided into four groups: Caucasian, European, Asian, and Middle Eastern. The *TGF-β1* 29T>C polymorphism was found to be associated with BC risk in the heterozygous model of the Asian population (OR = 1.115, 95% CI = 1.006–1.237, *p* = 0.039). In contrast, the same polymorphism was associated with lower odds of BC in the heterozygous model of the Middle Eastern population (OR = 0.602, 95% CI = 0.375–0.966, *p* = 0.035) (Figure 5). No correlation was found between the *TGF-β1* 29T>C polymorphism and BC risk in the Caucasian and European populations. Similarly, results of other variants of *TGF-β1* polymorphism have shown no association with BC risk in ethnic groups. These findings suggested that the *TGF-β1* 29T>C polymorphism alone is associated with BC risk in the Asian population. The ORs and 95% CIs of all stratified ethnic group analyses are provided in Table 2.

### 2.7. Sensitivity Analysis

One-way sensitivity analysis was performed to analyze the effect of each study on the combined OR. During the sensitivity analysis for each polymorphism, each relevant study was deleted iteratively from the dataset prior to the analysis. No single study had a significant influence on any of the combined ORs, suggesting that our meta-analysis is relatively robust and credible (Supplementary Figures S5–S7). 

### 2.8. Trial Sequential Analysis (TSA)

TSA was used to investigate whether the number of samples included in the present study was sufficient for detecting a possible role of each *TGF-β1* polymorphism in BC. The Z curve touched the trial monitoring boundaries or reached the required information line, indicating that a sufficient number of studies were included in the meta-analysis for overall and ethnic group investigations (Supplementary Figure S8).

## 3. Discussion 

The *TGF-β1* signaling pathway has been studied extensively in several cancers, including BC. *TGF-β1* plays an important role in cell differentiation, migration, invasion, and tumor growth [4]. Both tumorigenic and tumor-suppressive roles of *TGF-β1* have been well established [5]. Elevated plasma levels of *TGF-β1* have been associated with cancer development, and *TGF-β1* polymorphisms have been found to cause high transcription and expression of *TGF-β1* [9,11]. The prominent role of *TGF-β1* in cancer progression underscores the importance of studying the association between *TGF-β1* polymorphisms and BC risk. 

The most common *TGF-β1* polymorphism is a substitution of cytosine with thymine at the 29th nucleotide (*TGF-β1* 29 T>C), which results in the substitution of the proline at codon 10 in exon 1 with leucine [8,9,10,11]. *TGF-β1* 29T>C has been associated with elevated levels of *TGF-β1* in plasma [9]. Studies have also suggested that *TGF-β1* 29T>C is associated with BC risk; however, contrasting results were reported. Dunning et al. suggested that the *TGF-β1* 29T>C polymorphism is associated with BC risk; they also found that this polymorphism is associated with elevated levels of the *TGF-β1* protein [9]. In contrast, Ziv et al. reported no such associations [12]. Marchand et al. reported similar results in their multi-ethnic study, which suggested no association between the *TGF-β1* 29T>C polymorphism and BC [13]. Interestingly, Lee et al. demonstrated that the *TGF-β1* 29T>C polymorphism was associated with an increased risk of BC in postmenopausal women [32,33]. However, Hishida et al. observed a negative correlation between the *TGF-β1* 29T>C polymorphism and BC risk in premenopausal women [27]. Interestingly, Shin et al. demonstrated that no association was found in the initial stages of BC, whereas a significant correlation was observed in later stages [30].

A systematic meta-analysis was performed to provide conclusive evidence for the association of TGF-β polymorphism with breast cancer risk. Interestingly, neither the overall allele nor genotypic models showed an association between the *TGF-β1* 29T>C polymorphism and BC risk. However, the *TGF-β1* 29T>C polymorphism was found to be associated with an increased risk of BC in the heterozygous model of the Asian population. In contrast, the *TGF-β1*29T>C polymorphism favors lower odds of BC in the heterozygous model of the Middle Eastern population. 

The *TGF-β1*-509 C/T polymorphism is located in the promoter region of *TGF-β1*. Recent studies demonstrated the association between the *TGF-β1*-509 C/T polymorphism and elevated levels of the TGF-β protein. It has also been well established that the *TGF-β1*-509 C/T polymorphism is highly associated with BC risk. Both Dunning et al. [9] and Parvizi et al. [52] suggested that the polymorphism is associated with increased risk of invasive BC, whereas Cox et al. showed no such association [35]. Vinod et al. demonstrated that heterozygosity of the *TGF-β1*-509 C/T polymorphism was associated with BC susceptibility in an Indian population [51]. However, Babyshkina et al. reported that homozygosity of the T allele of the *TGF-β1*-509 C/T polymorphism was not associated with BC in a Russian population [49]. These inconclusive and controversial results suggest the necessity of a cumulative and robust analysis to arrive at a definitive conclusion regarding the association between this polymorphism and cancer risk in multiple ethnic groups. Six meta-analyses were performed to address these issues; three investigated the association between the *TGF-β1*-509 C/T polymorphism and multiple cancer subtypes. Huang et al., Qi et al., and Woo et al. performed meta-analyses examining the association between the *TGF-β1*-509 C/T polymorphism and BC specifically [14,17,58]. However, these studies were performed using a limited number of studies, and Huang et al. included a study that was not related to BC risk [58]. These drawbacks can influence the analysis and affect the results. In order to definitively determine the association between the *TGF-β1*-509 C/T polymorphism and BC, we performed a meta-analysis including additional studies and excluding studies not related to BC risk. Our robust analysis demonstrated that the *TGF-β1*-509 C/T polymorphism is not associated with BC in the overall population or within specific ethnic groups.

Researchers have identified a polyalanine variant in exon 5 of *TGF-β1* and analyzed its association with BC risk. Recent studies have also suggested that the TGF-β1*6A polymorphism is associated with BC risk. Specifically, Kaklamani et al., Pasche et al., and Song et al. reported that the polymorphism is associated with an increased incidence of BC [19,31,55]. Pasche et al. also found that the TGF-β1*6A polymorphism is associated with risk for other cancers, including colorectal and ovarian cancer [19]. However, Chen et al., Colleran et al., and Cox et al. suggested that the TGF-β1*6A variant is not associated with BC risk. Meta-analyses have been performed; however, they investigated the association between the TGF-β1*6A polymorphism and all cancer types; no meta-analyses have yet correlated this polymorphism with BC specifically [20,59]. Thus, in the present study, we analyzed for the first time the association between the TGF-β1*6A polymorphism and BC risk. Our cumulative analysis suggests that the TGF-β1*6A variant is not associated with BC risk. In our analysis of the European ethnic group, the TGF-β1*6A variant also showed no association with BC risk.

Although various meta-analyses have already been reported in the literature, the present study has several advantages over them. A recent investigation by Alqumber et al. included reports published until the year 2013; in contrast, we screened for studies published up to May 2019, resulting in the inclusion of three additional studies. Furthermore, we assessed the possible association of three *TGF-β1* polymorphisms (*TGF-β1* 29>T/C, *TGF-β1*-509 C/T, and TGF-β1*6A) with the risk of BC [60]. However, multiple comparisons failed to show a significant correlation between these polymorphisms with BC risk: the present study warrants the non-importance of TGF-β1 polymorphisms on the pathogenesis of BC. Finally, TSA revealed that our meta-analysis included a sufficient number of studies, performed worldwide, to dissect the possible association of *TGF-β1* variants with BC risk, and further investigation is not required.

## 4. Materials and Methods

### 4.1. Literature Search Strategy and Selection of Relevant Studies

Three researchers independently conducted a comprehensive systematic electronic literature search using the online databases Pubmed, EMBASE, and Google Scholar. The following terms were used either alone or in combination: “Transforming Growth Factor β1 polymorphism”; “breast cancer”; for the *TGF-β1* 29T>C polymorphism: “*TGF-β1* 29T>C”, “*TGF-β1*Pro10Leu”, “*TGF-β1*T869C”, “rs1982073”, “rs180047029”; for the *TGF-β1*-509 C/T polymorphism: “*TGF-β1*-509 C/T”, “*TGF-β1* 1349C>T”, “rs1800469”; and for the TGF-β1*6A polymorphism: “TGF-β1*6A”, “rs11466445”. We also examined the cross-references in the retrieved studies for publications that were missed in the above search. 

### 4.2. Criteria for the Inclusion and Exclusion of Studies 

The present meta-analysis included studies that evaluated the association between *TGF-β1* polymorphisms and BC risk, were published in English, presented original data, and provided the genotypic frequency of both case and control samples or had odds ratios (ORs) with 95% confidence interval (CI) values. The analysis excluded reviews, abstracts, duplicate or overlapping studies, studies not published in English, studies that correlated *TGF-β1* polymorphisms with non-breast cancers, and studies that did not provide genotypic or allele frequencies for both case and control samples. 

### 4.3. Data Extraction

Each publication was assessed thoroughly, and data were extracted independently by three researchers, following the same pattern and extracting the same data essential for meta-analyses that have been previously described [22,23]. The researchers conducted group discussions to resolve any discrepancies in extraction. 

### 4.4. Statistical Analysis

Statistical analysis was performed using Comprehensive Meta-Analysis Software (CMA version 3) (Biostat, 14 North Dean Street, Englewood, NJ 07631 USA). In order to appraise the associations between *TGF-β1* polymorphisms and BC risk, the combined OR, 95% CIs, and their respective *p*-values were calculated by comparing the cancer patient samples vs. respective healthy controls. Heterogeneity among the studies and publication bias were calculated using Begg’s funnel plots and chi-squared-based Cochran’s Q tests, respectively. Egger’s regression tests were performed to analyze and measure the symmetry of funnel plots, as described previously [22]. Asymmetrical funnel plots were made symmetrical using the “Trim and Fill” method. Random models were used to calculate the combined ORs for studies showing heterogeneity [24]. In contrast, fixed models were used to calculate the combined ORs for studies with homogeneity [25]. 

### 4.5. Trial Sequential Analysis (TSA)

An appropriate meta-analysis must include all eligible studies published to date to draw definitive conclusions. The number of studies available in the literature in the studied areas should be sufficient for decisive concluding remarks. TSA is a statistical tool developed by the Copenhagen Trial Unit, Centre for Clinical Intervention Research, Denmark, to estimate the sample size required to reach significance with definitive power. In TSA, a Z curve analysis is performed to check whether a sufficient number of samples is included in the study. If the Z curve intersects the TSA monitoring boundary before reaching the required information size or if the total number of samples exceeds the required information line, then the total number of studies included in the investigation is sufficient, and additional trials are not required. On the other hand, if the Z curve fails to touch the TSA monitoring boundaries or cross the required information line, the number of studies is limited and more are required to draw any definitive conclusions. TSA software version 0.9 (http://www.ctu.dk/tsa) was used for this analysis.

## 5. Conclusions

TGF-β1 plays an important role in BC progression, metastasis, stemness, and chemo-resistance. TGF-β1 has both pro-oncogenic and tumor-suppressive roles during cancer development. High levels of TGF-β1, which are influenced by *TGF-β1* polymorphisms, have been associated with cancer risk. *TGF-β1*polymorphisms have been associated with BC risk, but conflicting results have also been reported recently. Previous meta-analyses either included studies on *TGF-β1* polymorphisms that did not evaluate associations with BC or did not include new studies with huge datasets that could greatly influence their results. To address these shortcomings, a stringent and highly robust analysis was conducted to provide conclusive evidence for the association between *TGF-β1* polymorphisms and BC risk. The current meta-analysis included large datasets from all recent eligible studies and excluded studies that did not evaluate the associations between these polymorphisms and BC. This study suggests that *TGF-β1* polymorphisms are not associated with BC risk in the overall population. Both *TGF-β1*-509 C/T and TGF-β1*6A also showed no association with BC risk in stratified analyses. However, the *TGF-β1* 29T>C polymorphism was found to be associated with BC risk in the Asian ethnic group.

## Figures and Tables

**Table 1:** Characteristics of all studies included for meta-analysis.

**S.No.**	**First Author**	**Year**	**Ethnicity**	**Case**	**Control**	**Genotype Distribution**						**Allele Distribution (%)**				**Genotyping method**
**TGFβ1 29C>T (Pro10Leu) or T869C or rs1982073 or rs1800470**						**Breast cancer**			**Control**			**Breast Cancer**		**Control**		
						**TT**	**TC**	**CC**	**TT**	**TC**	**CC**	**T**	**C**	**T**	**C**	
1	Ziv et al [12]	2001	Caucasian	146	2929	56	80	10	1068	1413	448	192 (65.75)	100 (34.25)	3549 (60.58)	2309 (39.42)	PCR-RFLP
2	Krippl et al [26]	2003	Caucasian	495	499	196	219	80	182	229	88	611 (61.71)	379 (38.29)	593 (59.42)	405 (40.58)	PCR
3	Dunning et al ^a^ [9]	2003	UK	1,752	1877	99	1228	425	735	889	253	1426 (40.7)	2078 (59.3)	2359 (62.84)	1395 (37.16)	Taqman
4	Dunning et al ^b^ [9]	2003	European	415	574	116	220	79	202	274	98	452 (54.45)	378 (45.54)	678 (59.06)	470 (40.94)	Taqman
5	Dunning et al ^c^ [9]	2003	Finnland	481	451	255	191	35	232	191	28	701 (72.90)	261 (27.1)	655 (72.62)	247 (27.38)	Taqman
6	Hishida et al [27]	2003	Asian	232	177	67	107	58	42	87	48	241 (51.9)	223 (48.1)	171 (48.30)	183 (51.7)	PCR-CTPP
7	Marchand et al ^a^ [13]	2004	Afro-American	233	612	76	112	45	205	289	118	264 (56.65)	202 (43.35)	699 (57.11)	525 (42.89)	Taqman
8	Marchand et al ^b^ [13]	2004	Latina	225	647	67	111	47	179	308	160	245 (54.44)	205 (45.56)	666 (51.47)	628 (48.53)	Taqman
9	Marchand et al ^c^ [13]	2004	White	299	402	114	137	48	141	183	78	365 (61.0)	233 (39)	465 (57.84)	339 (42.16)	Taqman
10	Marchand et al ^d^ [13]	2004	Japanese	303	385	62	163	78	91	183	111	287 (47.36)	319 (52.64)	365 (47.40)	405 (52.6)	Taqman
11	Marchand et al ^e^ [13]	2004	Hawaiian	63	268	19	27	17	74	140	54	65 (51.6)	61 (48.4)	288 (53.73)	248 (46.27)	Taqman
12	jin et al ^a^ [28]	2004	Finnland	223	234	119	93	11	123	91	20	331 (74.2)	115 (25.8)	337 (72.0)	131 (28.0)	PCR-RFLP, PCR-ALF
13	jin et al ^b^ [28]	2004	Polish	415	205	151	189	75	66	105	34	491 (59.15)	339 (40.85)	237 (57.8)	173 (42.2)	PCR-RFLP, PCR-ALF
14	jin et al ^c^ [28]	2004	German	160	162	63	72	25	46	96	20	198 (61.87)	122 (38.13)	188 (58.02)	136 (41.98)	PCR-RFLP, PCR-ALF
15	jin et al ^d^ [28]	2004	Swedish	84	173	31	38	15	70	77	26	100 (59.5)	68 (40.5)	217 (62.72)	129 (37.28)	PCR-RFLP, PCR-ALF
16	Saha et al [29]	2004	Asian	23	84	10	13	0	47	33	4	33 (71.7)	13 (28.3)	127 (75.6)	41 (24.4)	PCR-SSCP
17	Shin et al [30]	2005	Asian	1,114	1189	258	554	302	255	615	319	1070 (48.1)	1158 (51.9)	1125 (47.31)	1253 (52.69)	PCR-RFLP
18	Kaklamani et al [31]	2005	Caucasian	658	841	200	339	119	240	419	182	739 (56.1)	577 (43.9)	899 (53.45)	783 (46.55)	PCR-seq
19	Lee et al [32]	2005	Asian	558	501	135	288	135	148	235	118	558 (50)	558 (50)	531 (53.0)	471 (47.0)	PCR-CTPP
20	Feigelson et al [33]	2006	Caucasian	485	481	182	233	70	181	221	79	597 (61.55)	373 (38.45)	583 (60.60)	379 (39.40)	Taqman
21	Scola et al [34]	2006	Caucasian	84	106	41	27	16	35	52	19	109 (64.88)	59 (35.12)	122 (57.55)	90 (42.45)	PCR-RFLP
22	D Cox et al [35]	2007	Caucasian	1,185	1651	469	548	168	613	797	241	1486 (62.7)	884 (37.3)	2023 (61.27)	1279 (38.73)	Taqman
23	Gonullu et al [36]	2007	Turkey	38	24	20	10	8	11	9	4	50 (65.8)	26 (34.2)	31 (64.58)	17 (35.42)	PCR
24	A Cox et al (GESBC) [37]	2007	Caucasian	556	713	169	284	103	255	338	120	622 (55.9)	490 (44.1)	848 (59.47)	578 (40.53)	Taqman
25	A Cox et al (HBCS) [37]	2007	Caucasian	1,073	1013	386	506	181	388	471	154	1278 (59.55)	868 (40.45)	1247 (61.55)	779 (38.45)	Taqman
26	A Cox et al (IARC-Thai) [37]	2007	Asian	453	356	189	213	51	161	162	33	591 (65.23)	315 (34.77)	484 (67.98)	228 (32.02)	Taqman
27	A Cox et al (Kuopio) [37]	2007	Caucasian	435	442	229	175	31	225	189	28	633 (72.76)	237 (27.24)	639 (72.29)	245 (27.71)	Taqman
28	A Cox et al (Mayo Clinic) [37]	2007	Caucasian	793	837	296	404	93	307	409	121	996 (62.8)	590 (37.2)	1023 (61.1)	651 (38.89)	Taqman
29	A Cox et al (PBCS) [37]	2007	Caucasian	1,841	2254	617	890	334	797	1104	353	2124 (57.67)	1558 (42.33)	2698 (59.85)	1810 (40.15)	Taqman
30	A Cox et al (SEARCH) [37]	2007	Caucasian	4,504	5689	1670	2138	696	2200	2716	773	5478 (60.8)	3530 (39.2)	7116 (62.54)	4262 (37.46)	Taqman
31	A Cox et al (Seoul) [37]	2007	Asian	643	529	162	327	154	155	253	121	651 (50.62)	635 (49.38)	563 (53.21)	495 (46.79)	Taqman
32	A Cox et al (SASBAC) [37]	2007	Caucasian	1,303	1494	539	596	168	657	637	200	1674 (64.2)	932 (35.8)	1951 (65.3)	1037 (34.7)	Taqman
33	A Cox et al (CNIO) [37]	2007	Caucasian	640	739	160	313	167	217	355	167	633 (49.45)	647 (50.55)	789 (53.38)	689 (46.62)	Taqman
34	A Cox et al (USRT) [37]	2007	Caucasian	705	1043	243	339	123	382	494	167	825 (58.51)	585 (41.49)	1258 (60.31)	828 (39.69)	Taqman
35	Rajkumar et al [38]	2008	Asian	250	500	80	126	44	190	234	76	286 (57.2)	214 (42.8)	614 (61.4)	386 (38.6)	PCR-CTPP
36	Joshi et al [39]	2011	Asian	203	384	67	104	32	114	181	89	238 (58.62)	168 (41.38)	409 (53.25)	359 (46.75)	PCR-SSP
37	Zhang et al [40]	2011	Asian	170	178	38	76	56	49	79	50	152 (44.7)	188 (55.3)	177 (49.72)	179 (50.28)	PCR-RFLP
38	Pooja et al [41]	2013	Asian	465	239	85	165	214	51	123	64	335 (36.1)	593 (63.9)	225 (47.27)	251 (52.73)	PCR-seq
39	Eva et al [42]	2013	Caucasian	274	252	99	131	44	87	117	48	329 (60.0)	219 (40)	291 (57.74)	213 (42.26)	Taqman
40	Amani et al [43]	2014	Iranian	100	104	45	28	27	33	41	30	118 (59)	82 (41)	107 (51.44)	101 (48.56)	PCR-seq
41	Joshi et al [44]	2014	Asian	172	229	60	81	31	61	114	54	201 (58.43)	143 (41.57)	236 (51.53)	222 (48.47)	PCR-SSP
42	Tezerjani et al [45]	2015	Iranian	60	60	18	29	13	16	32	12	65 (54.17)	55 (45.83)	64 (53.33)	56 (46.67)	PCR-ARMS
**S.No**	**First Author**	**Year**	**Ethnicity**	**Case**	**Control**	**Genotype Distribution**						**Allele Distribution (%)**				**Genotyping method**
	**TGFβ1 -509 C/T or -1349C>T or rs1800469**					**Breast Cancer**			**Control**			**Breast Cancer**		**Control**		
						**CC**	**CT**	**TT**	**CC**	**CT**	**TT**	**C**	**T**	**C**	**T**	
1	Dunning et al ^a^ [9]	2003	UK	2439	2366	1181	1014	244	1194	977	195	3376 (69.21)	1502 (30.79)	3365 (71.11)	1367 (28.89)	Taqman
2	Dunning et al ^b^ [9]	2003	European	417	634	159	201	57	281	287	66	519 (62.23)	315 (37.77)	849 (66.96)	419 (33.04)	Taqman
3	Dunning et al ^c^ [9]	2003	Finnland	480	452	277	176	27	252	177	23	730 (76.04)	230 (23.96)	681 (75.33)	223 (24.67)	Taqman
4	jin et al ^a^ [28]	2004	Finnland	221	320	133	80	8	182	119	19	346 (78.28)	96 (21.72)	483 (75.47)	157 (24.53)	PCR-RFLP, PCR-ALF
5	jin et al ^b^ [28]	2004	Polish	170	188	71	81	18	74	95	19	223 (65.59)	117 (34.41)	243 (64.63)	133 (35.37)	PCR-RFLP, PCR-ALF
6	Saha et al [29]	2004	Asian	26	97	3	22	1	35	53	9	28 (53.85)	24 (46.15)	123 (63.4)	71 (36.6)	PCR-SSCP
7	Shin et al [30]	2005	Asian	1118	1206	260	559	299	260	628	318	1049 (48.26)	1157 (51.74)	1148 (47.6)	1264 (52.4)	PCR-RFLP
8	D Cox et al [35]	2007	Caucasian	1195	1663	600	506	89	786	723	154	1706 (71.38)	684 (28.62)	2295 (69)	1031 (31)	Taqman
9	Jakubowska et al [46]	2009	Caucasian	1011	1068	454	451	106	465	476	127	1359 (67.21)	663 (32.79)	1406 (65.82)	730 (34.18)	PCR-RFLP
10	Jakubowska et al [47]	2010	Caucasian	319	290	127	144	48	115	145	30	398 (62.38)	240 (37.62)	375 (64.66)	205 (35.34)	PCR-RFLP
11	MARIE-GENICA [48]	2010	Caucasian	3146	5485	1529	1315	302	2616	2313	556	4373 (69.5)	1919 (30.5)	7545 (68.78)	3425 (31.22)	MALDI-TOF Msa and PCR-RFLP
12	Babyshkina et al [49]	2011	Russian	218	290	89	108	21	103	133	54	286 (65.6)	150 (34.4)	339 (58.45)	241 (41.55)	PCR-RFLP
13	Zhang et al [40]	2011	Asian	170	178	28	93	49	41	84	53	149 (43.82)	191 (56.18)	166 (46.63)	190 (53.37)	PCR-RFLP
14	Quan et al ^a^ [50]	2013	Afro-American	454	405	268	164	22	215	165	25	700 (77.09)	208 (22.91)	595 (73.46)	215 (26.54)	MassArray IPLEX Gold Assay
15	Quan et al ^b^ [50]	2013	European	327	312	124	147	56	134	135	43	395 (60.4)	259 (39.6)	403 (64.58)	221 (35.42)	MassArray IPLEX Gold Assay
16	Vinod et al [51]	2013	Asian	153	128	64	66	23	70	36	22	194 (63.4)	112 (36.6)	176 (68.75)	80 (31.25)	PCR-ARMS
17	Parvizi et al [52]	2016	Iranian	100	100	31	50	19	21	48	31	112 (56)	88 (44)	90 (45)	110 (55)	PCR-RFLP
**S.No**	**First Author**	**Year**	**Ethinic group**	**Case**	**Control**	**Genotype Distribution**						**Allele Distribution (%)**				**Genotyping method**
	TGFβ1* 6A or rs11466445					**Case**			**Control**			**Case**		**Control**		
						**9A/9A**	**9A/6A**	**6A/6A**	**9A/9A**	**9A/6A**	**6A/6A**	**9A**	**6A**	**9A**	**6A**	
1	Pasche et al [19]	1999	USA	152	732	128	24	0	654	78	0	280 (92.11)	24 (7.89)	1386 (94.67)	78 (5.33)	PCR-RFLP
2	Baxter et al [21]	2002	United Kingdom	355	248	268	83	4	207	39	2	619 (87.18)	91 (12.82)	453 (91.33)	43 (8.67)	PCR-SSCP
3	Reiss et al [53]	2004	USA	98	91	87	11	0	77	14	0	185 (94.39)	11 (5.61)	168 (92.31)	14 (7.69)	PCR-RFLP
4	Caldes et al [53]	2004	USA	506	292	397	106	3	250	42	0	900 (88.93)	112 (11.07)	542 (92.81)	42 (7.19)	PCR-RFLP
5	Offit et al [53]	2004	USA	462	330	391	67	4	291	38	1	849 (91.88)	75 (8.12)	620 (93.94)	40 (6.06)	PCR-RFLP
6	jin et al ^a^ [28]	2004	Finnish	221	234	177	38	6	171	60	3	392 (88.69)	50 (11.31)	402 (85.9)	66 (14.1)	PCR-RFLP
7	jin et al ^b^ [28]	2004	Polish	170	202	140	28	2	176	26	0	308 (90.59)	32 (9.41)	378 (93.56)	26 (6.44)	PCR-RFLP
8	Kaklamani et al [31]	2005	USA	611	690	515	92	4	612	77	1	1122 (91.82)	100 (8.18)	1301 (94.28)	79 (5.72)	PCR-seq
9	Chen et al [54]	2006	USA	115	130	92	23	0	111	18	1	207 (90)	23 (10)	240 (92.31)	20 (7.69)	PCR-SSCP
10	Figelson et al [33]	2006	USA	387	384	387			384							PCR
11	D Cox et al [35]	2007	USA	1,187	1673	968	207	12	1352	302	19	2143 (90.27)	231 (9.73)	3006 (89.84)	340 (10.16)	Taqman
12	Song et al [55]	2007	Sweden	763	852	598	152	13	682	160	10	1348 (88.34)	178 (11.66)	1524 (89.44)	180 (10.56)	PCR
13	Colleran et al [56]	2010	Ireland	960	958	796	154	10	785	160	13	1746 (90.94)	174 (9.06)	1730 (90.29)	186 (9.71)	PCR
14	Jakubowska et al [47]	2010	Poland	282	252	282			252							PCR-RFLP
15	Joshi et al [39]	2011	Asian	209	391	196	12	1	361	28	2	404 (96.65)	14 (3.35)	750 (95.91)	782 (4.09)	PCR-SSP
16	kamali et al [57]	2015	Iranian	280	280	251	25	4	241	27	12	525 (94.09)	33 (5.91)	509 (90.89)	560 (9.11)	PCR

Dunning et al. UK population designated as ^(a)^, European ethnic group designated as ^(b)^, and Finland population designated as ^(c)^. Marchand et al. African-American ethnic group designated as ^(a)^, Latinos designated as ^(b)^, Whites designated as ^(c)^, Japanese designated as ^(d)^, and Hawaiian designated as ^(e)^. Jin et al. Finland population designated as ^(a)^, Polish designated as ^(b)^, German designated as ^(c)^, and Swedish designated as ^(d)^. Quin et al. African-American ethnic group designated as ^(a)^ and European designated as ^(b)^.

**Table 2:** Stratified analysis of TGFβ polymorphisms and their association with BC risk.

	Model	Odds Ratio (OR)	95% CI	*p*-Value
***TGF-β1* 29C>T (Pro10Leu) or T869C or rs1982073 or rs1800470**				
**Caucasian**				
1	Overall allele C vs. T	1.008	0.961–1.057	0.749
2	Homozygous CC vs. TT	1.007	0.907–1.120	0.891
3	Heterozygous TC vs. TT	1.038	0.990–1.088	0.123
4	Recessive CC vs. TT + TC	0.997	0.910–1.093	0.947
5	Dominant CC + TC vs. TT	1.039	0.994–1.086	0.093
**Asian**				
6	Overall allele C vs. T	1.058	0.952–1.176	0.298
7	Homozygous CC vs. TT	1.084	0.894–1.314	0.413
8	Heterozygous TC vs. TT	1.115	1.006–1.237	0.039
9	Recessive CC vs. TT + TC	1.055	0.875–1.271	0.576
10	Dominant CC + TC vs. TT	1.077	0.980–1.182	0.123
**European**				
11	Overall allele C vs. T	1.142	0.761–1.715	0.521
12	Homozygous CC vs. TT	1.466	0.516–4.116	0.473
13	Heterozygous TC vs. TT	1.319	0.536–3.247	0.547
14	Recessive CC vs. TT + TC	1.227	0.882–1.707	0.224
15	Dominant CC + TC vs. TT	1.355	0.552–3.312	0.505
**Middle Eastern**				
16	Overall allele C vs. T	0.833	0.625–1.110	0.212
17	Homozygous CC vs. TT	0.784	0.461–1.332	0.367
18	Heterozygous TC vs. TT	0.602	0.375–0.966	0.035
19	Recessive CC vs. TT + TC	1.011	0.632–1.618	0.964
20	Dominant CC + TC vs. TT	0.669	0.438–1.020	0.062
***TGFβ-1*-509 C/T or -1349C>T or rs1800469**				
**Caucasian**				
21	Overall allele T vs. C	0.953	0.905–1.004	0.068
22	Homozygous TT vs. CC	0.904	0.803–1.018	0.096
23	Heterozygous CT vs. CC	0.957	0.891–1.028	0.228
24	Recessive TT vs. CC + CT	0.924	0.825–1.036	0.175
25	Dominant TT + CT vs. CC	0.947	0.885–1.031	0.112
**European**				
26	Overall allele T vs. C	1.016	0.900–1.148	0.795
27	Homozygous TT vs. CC	1.039	0.770–1.401	0.804
28	Heterozygous CT vs. CC	1.038	0.950–1.135	0.405
29	Recessive TT vs. CC + CT	1.026	0.782–1.346	0.853
30	Dominant TT + CT vs. CC	1.058	0.972–1.151	0.190
**Asian**				
31	Overall allele T vs. C	1.023	0.925–1.132	0.657
32	Homozygous TT vs. CC	1.000	0.813–1.230	0.990
33	Heterozygous CT vs. CC	1.633	0.894–2.986	0.111
34	Recessive TT vs. CC + CT	0.993	0.842–1.171	0.936
35	Dominant TT + CT vs. CC	1.460	0.886–2.408	0.138
*TGF-β* *6A or rs11466445				
**European**				
36	Overall allele 6A vs. 9A	1.095	0.878–1.364	0.421
37	Homozygous 6A/6A vs. 9A/9A	1.247	0.749–2.075	0.396
38	Heterozygous 6A/9A vs. 9A/9A	1.056	0.809–1.378	0.690
39	Recessive 6A/6A vs. 9A/9A + 9A/6A	1.249	0.751–2.077	0.391
40	Dominant 6A/6A + 9A/6A vs. 9A/9A	1.049	0.839–1.311	0.675

## Supplementary Materials

The following are available online at https://www.mdpi.com/2072-6694/12/2/471/s1, Figure S1: PRISMA flowchart: Search, screening and selection of eligible studies included in meta-analysis for evaluating the association of TGF-β polymorphisms with breast cancer risk. Figure S2: Funnel plot: Assessment of publication bias using Begg’s funnel plot and Egger’s regression test. Asymmetric Begg’s funnel plot was made symmetric using “Trim and Fill” method. Figure S3: Forest plot: Analyzing the influence of detection methods on association of TGF-β1 29T>C polymorphism with breast cancer risk. Studies were separated based on the methods used for the detection of polymorphism [(A) PCR-RFLP and (B) Taqman] and were analyzed individually. Figure S4: Forest plot: Studies were separated based on the methods used for the detection of TGF-β1 -509 C/T polymorphism [(A) PCR-RFLP and (B) Taqman] and were analyzed individually to assess its association with increased risk of breast cancer. Figure S5: Sensitivity analysis: One individual study of TGF-β1 29T>C was excluded each time and was analyzed to assess the effect of individual study on combined OR. Figure S6: Sensitivity analysis: Each study was assessed for the potential influence on combined OR by deleting single study each time and performing the analysis for TGF-β1 -509 C/T polymorphism. Figure S7: Sensitivity analysis of each study representing TGF-β *6A polymorphism in the meta-analysis by one study removal method. Figure S8: Trial sequence analysis of all the included studies. TSA figure for TGFβ-1 29T>C overall (A), Caucasian (B), Asian (C) showed enough number of samples enrolled for the analysis except for the American (D). TSA analysis for other two SNPs TGF-β1 -509 C/T (E) and TGF-β*6A (F) also revealed similar results. Table S1: Statistics for heterogeneity test.
