# Supplementary material for: Association of TGF-β1 Polymorphisms with Breast Cancer Risk: A Meta-Analysis of Case–Control Studies
Source: Cancers (Basel). 2020 Feb 18;12(2):471. doi: 10.3390/cancers12020471 (PMC7072663; doi:10.3390/cancers12020471)
Supplement: Supplementary file 1 [file cancers-12-00471-s001.zip › Supplementary data/cancers-688225-supp.docx]

**Table 1 Characteristics of all studies included for meta-analysis**

Dunning  *et al*. UK population designated as (a), European ethnic group designated as (b) and Finland population designated as (c). Marchand *et al*. African-American ethnic group designated as (a), Latinos designated as (b), Whites designated as (c) Japanese designated as (d) and Hawaiian designated as (e). jin *et al*. Finland population designated as (a), Polish designated as (b), German designated as (c) and Swedish designated as (d). Quin *et al*. African-American ethnic group designated as (a) and European designated as (b).
