# Supplementary material for: Association of TGF-β1 Polymorphisms with Breast Cancer Risk: A Meta-Analysis of Case–Control Studies
Source: Cancers (Basel). 2020 Feb 18;12(2):471. doi: 10.3390/cancers12020471 (PMC7072663; doi:10.3390/cancers12020471)
Supplement: Supplementary file 1 [file cancers-12-00471-s001.zip › Supplementary data/Supplementary Figure Legends.pdf]

Supplementary Figure 1 PRISMA flowchart: Search, screening, and selection of eligible studies included in the meta-analysis to evaluate the association between TGF-β polymorphisms and BC risk.

Supplementary Figure 2 Funnel plot: Assessment of publication bias using Begg's funnel plots and Egger's regression tests. Asymmetric Begg's funnel plots were made symmetric using the "Trim and Fill" method.

Supplementary Figure 3 Forest plot: Assessment of the influence of detection methods on the association between the TGF-β1 29T>C polymorphism and BC risk. Studies were separated for analysis based on the methods used to detect the polymorphism [(**A**) Taqman and (**B**) PCR-RFLP].

Supplementary Figure 4 Forest plot: Assessment of the influence of detection methods on the association between the TGF-β1 -509 C/T polymorphism and BC risk. Studies were separated for analysis based on the methods used to detect the polymorphism [(**A**) Taqman and (**B**) PCR-RFLP].

Supplementary Figure 5 Sensitivity analysis: In the analysis of the TGF-β1 29T>C polymorphism, each relevant study was excluded iteratively from the dataset prior to analysis to assess its effect on the combined OR.

Supplementary Figure 6 Sensitivity analysis: In the analysis of the TGF-β1 -509 C/T polymorphism, each relevant study was excluded iteratively from the dataset prior to analysis to assess its effect on the combined OR.

Supplementary Figure 7 Sensitivity analysis: In the analysis of the TGF-β*6A polymorphism, each relevant study was excluded iteratively from the dataset prior to analysis to assess its effect on the combined OR.

Supplementary Figure 8 Trial sequence analysis of all the included studies: TSA figure for TGFβ-1 29T>C overall (**A**), Caucasian (**B**), Asian (**C**) showed enough number of samples enrolled for the analysis except for the American (**D**). TSA analysis for other two SNPs TGF-β1 -509 C/T (**E**) and TGF-β*6A (**F**) also revealed similar results.
