# Supplementary material for: Association of TGF-β1 Polymorphisms with Breast Cancer Risk: A Meta-Analysis of Case–Control Studies
Source: Cancers (Basel). 2020 Feb 18;12(2):471. doi: 10.3390/cancers12020471 (PMC7072663; doi:10.3390/cancers12020471)
Supplement: Supplementary file 1 [file cancers-12-00471-s001.zip › Supplementary data/Supplementary Table 1.pdf]

Supplementary Table 1 Statistics for heterogeneity test

S. No	Model	Heterogeneity analysis			Fixed/Random
		Q-value	p heterogeneity	I^2 value	
	**TGF-β 29 T>C**				
1	Overall allele C *vs* T	377.095	0.000	89.127	Random
2	Homozygous CC *vs* TT	393.834	0.000	89.590	Random
3	Heterozygous TC *vs* TT	438.804	0.000	90.656	Random
4	Recessive CC *vs* TT + TC	125.209	0.000	67.255	Random
5	Dominant CC + TC *vs* TT	469.857	0.000	91.274	Random
	**TGF-β -509 CT**				
6	Overall allele T *vs* C	36.032	0.003	55.594	Random
7	Homozygous TT *vs* CC	35.169	0.004	54.505	Random
8	Heterozygous CT *vs* CC	26.879	0.043	40.474	Fixed
9	Recessive TT *vs* CC + CT	30.770	0.014	48.001	Fixed
10	Dominant TT + CT *vs* CC	31.987	0.010	49.979	Fixed
	**TGF-β *6A**				
11	Overall allele 6A *vs* 9A	30.857	0.004	57.870	Random
12	Homozygous 6A/6A *vs* 9A/9A	11.791	0.380	6.789	Fixed
13	Heterozygous 6A/9A *vs* 9A/9A	25.601	0.019	49.221	Fixed
14	Recessive 6A/6A *vs* 9A/9A + 9A/6A	11.535	0.400	4.640	Fixed
15	Dominant 6A/6A + 9A/6A *vs* 9A/9A	29.782	0.013	49.634	Fixed
