# Supplementary figures and images for: Association of TGF-β1 Polymorphisms with Breast Cancer Risk: A Meta-Analysis of Case–Control Studies
Source: Cancers (Basel). 2020 Feb 18;12(2):471. doi: 10.3390/cancers12020471 (PMC7072663; doi:10.3390/cancers12020471)

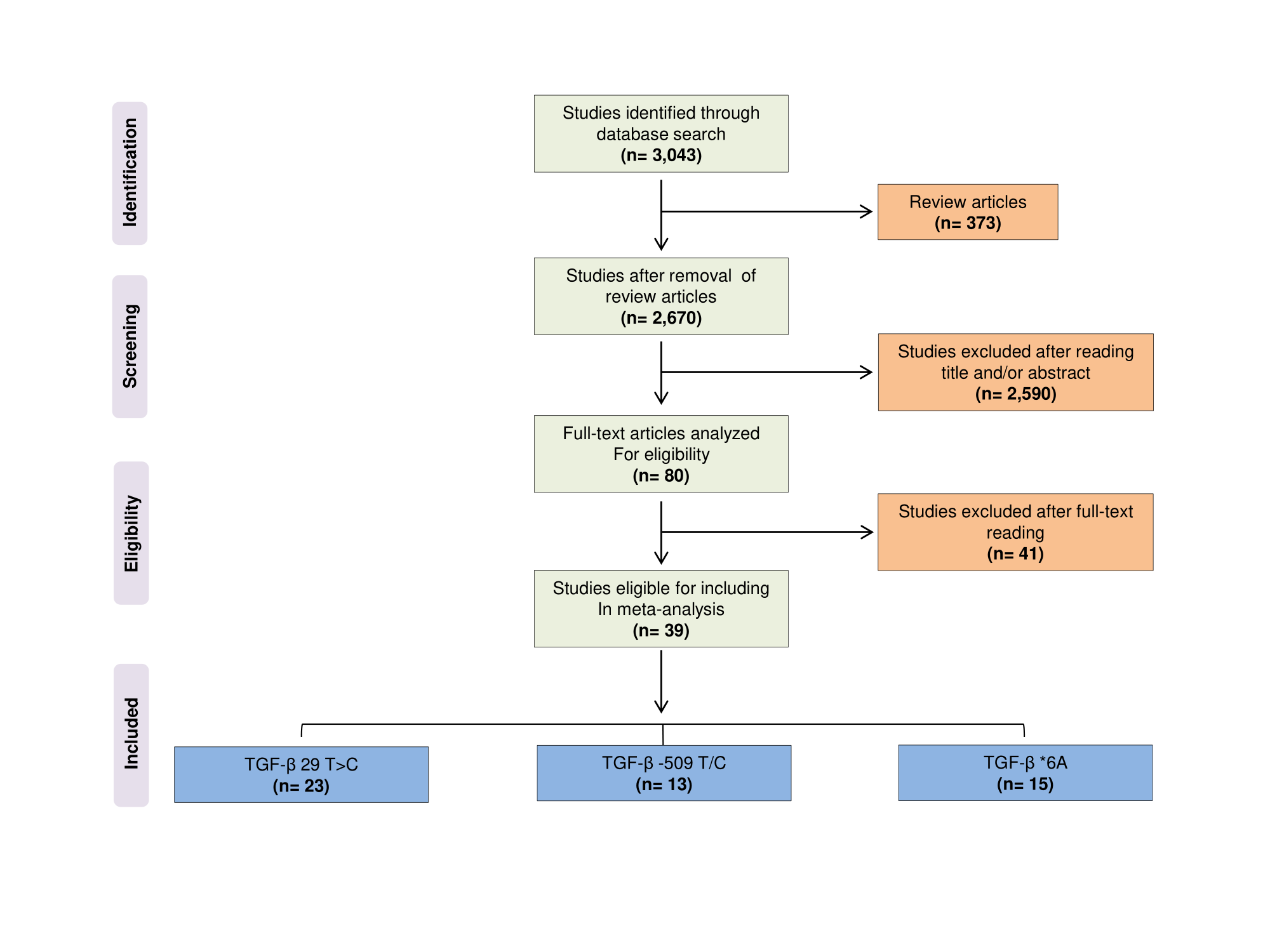

Supplement: Supplementary file 1 [file cancers-12-00471-s001.zip › Supplementary data/Supplementary Fig 1.tiff]

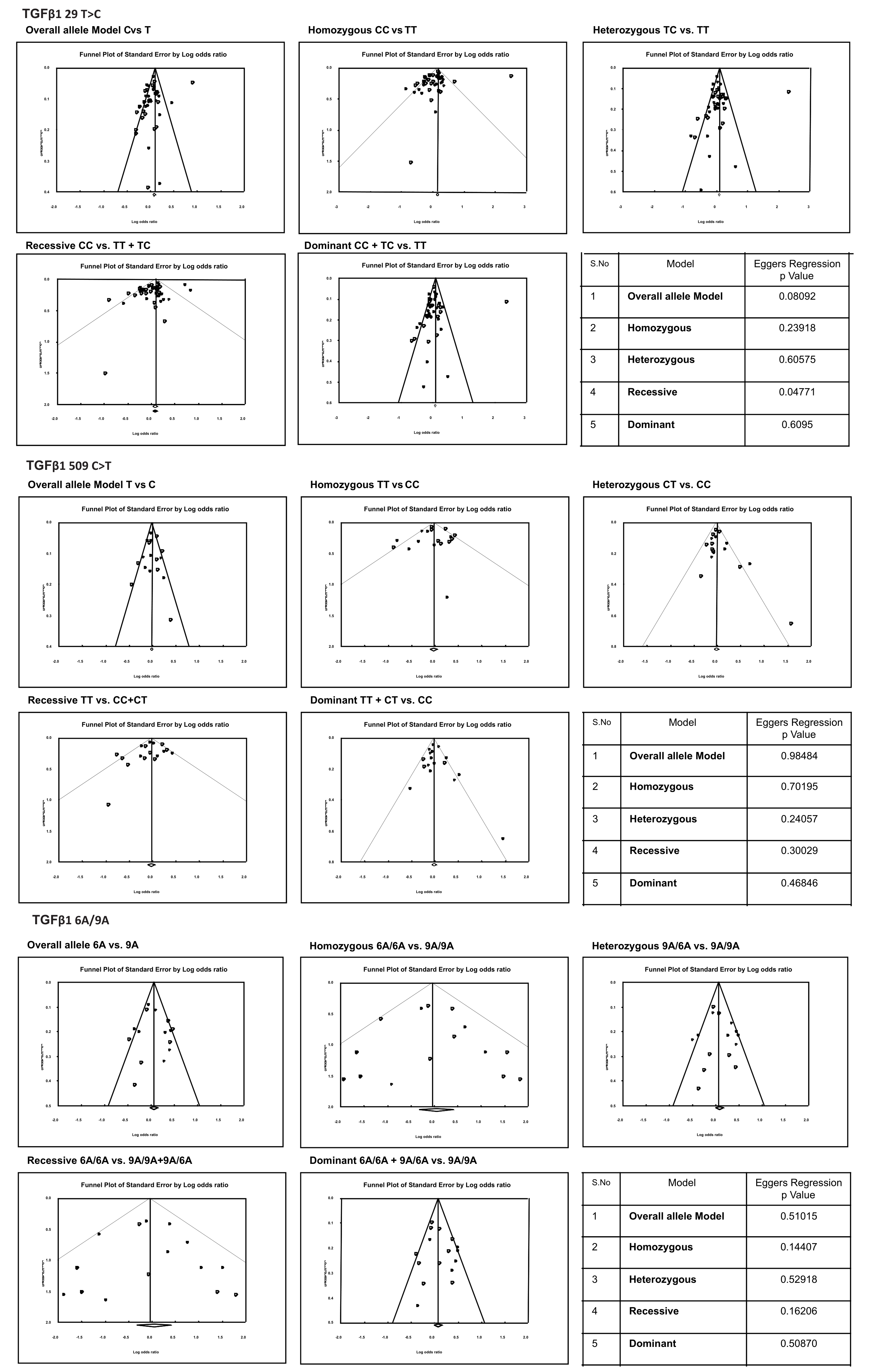

Supplement: Supplementary file 1 [file cancers-12-00471-s001.zip › Supplementary data/Supplementary Fig 2.tiff]

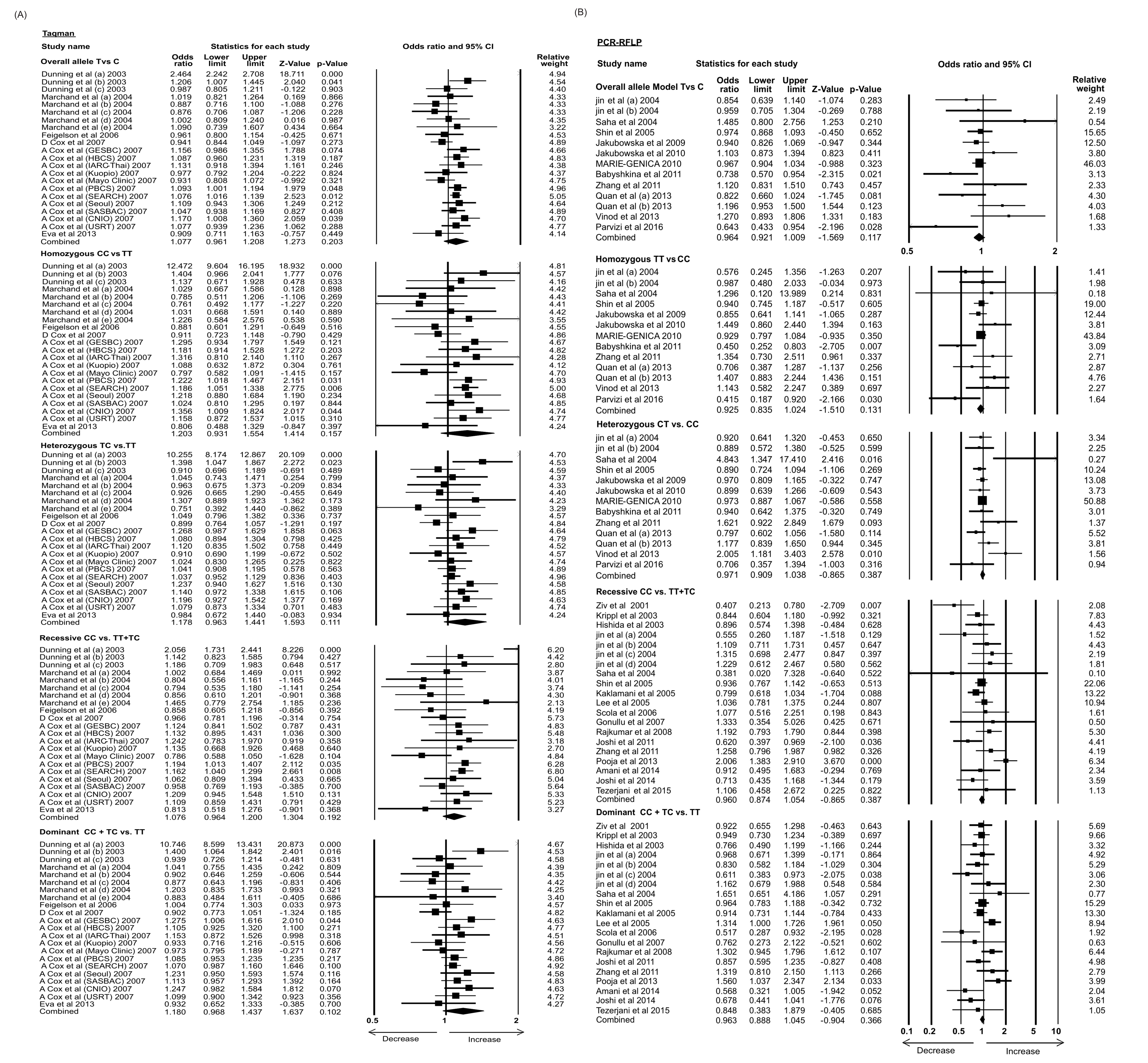

Supplement: Supplementary file 1 [file cancers-12-00471-s001.zip › Supplementary data/Supplementary Fig 3.tiff]

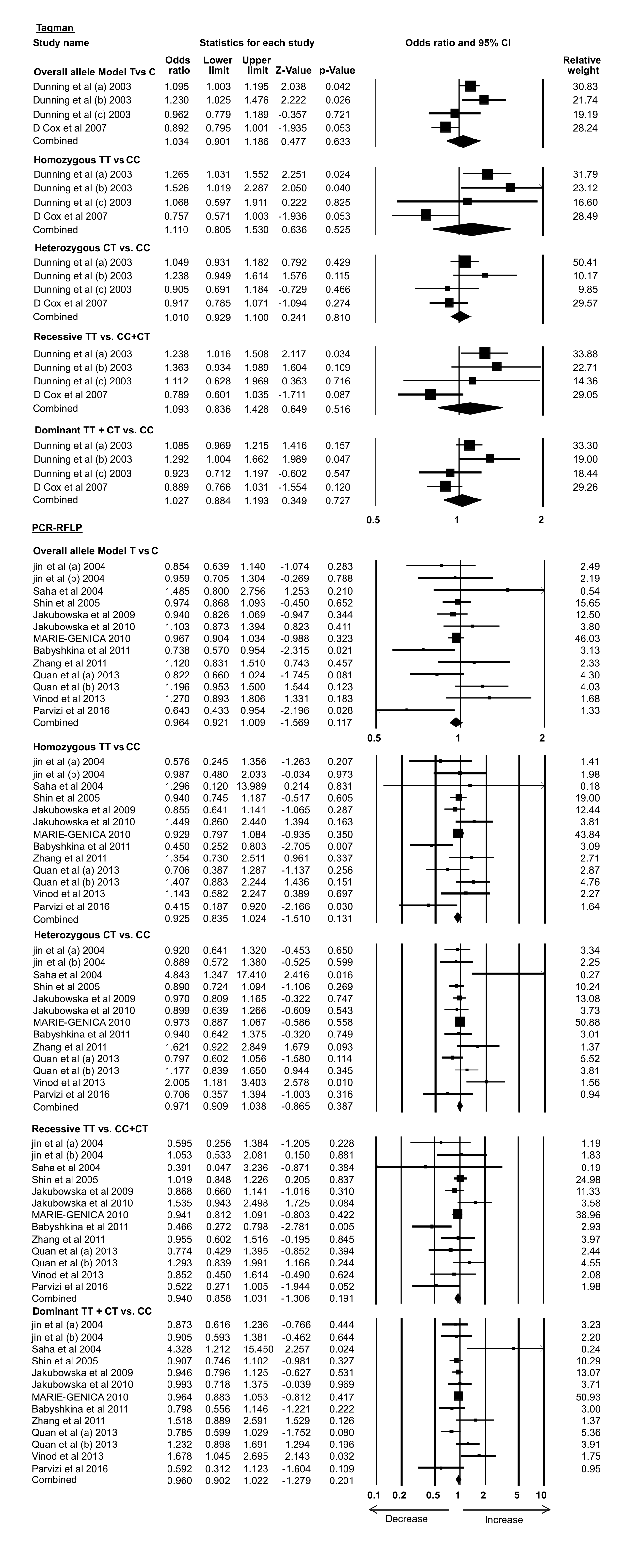

Supplement: Supplementary file 1 [file cancers-12-00471-s001.zip › Supplementary data/Supplementary Fig 4 .tiff]

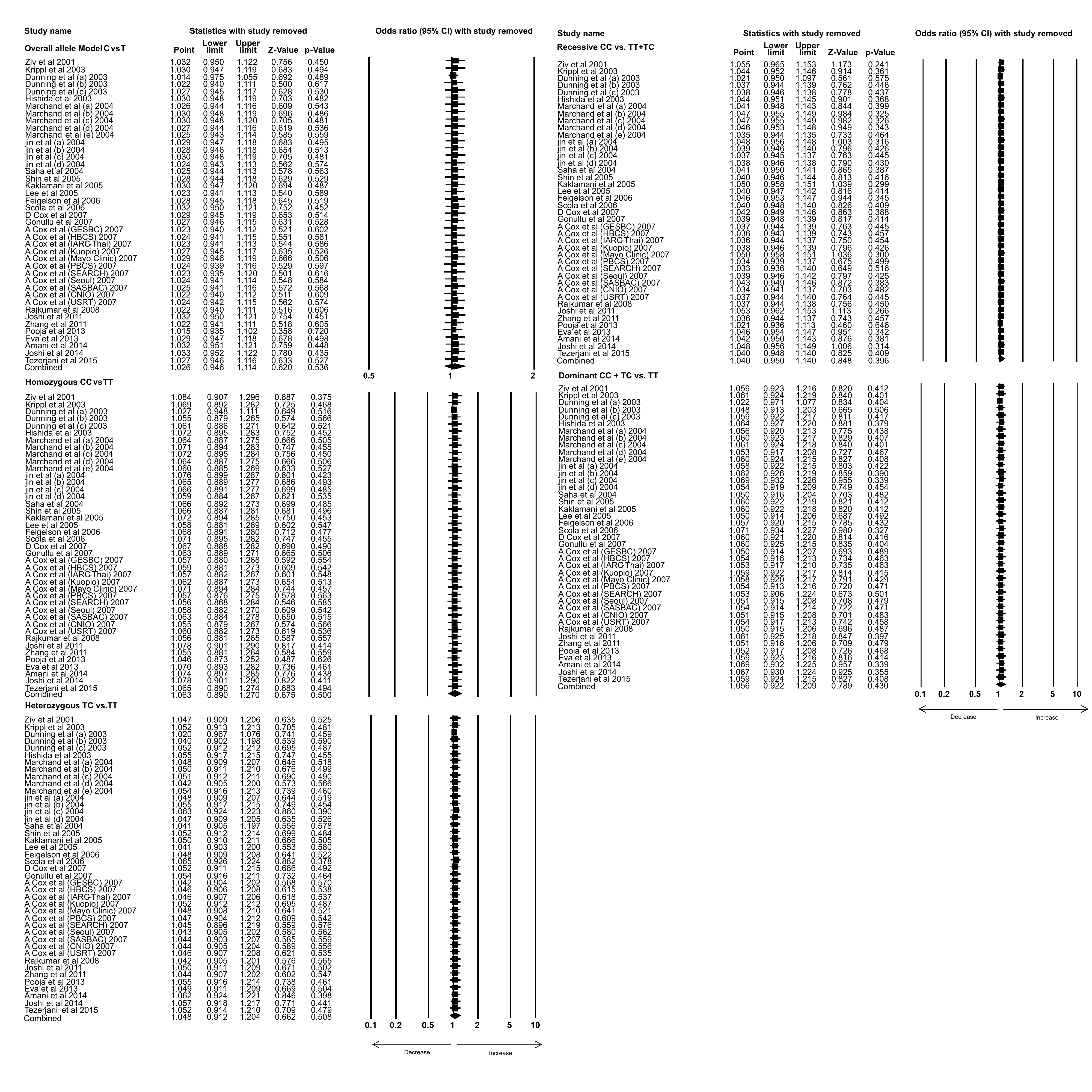

Supplement: Supplementary file 1 [file cancers-12-00471-s001.zip › Supplementary data/Supplementary Fig 5.tiff]

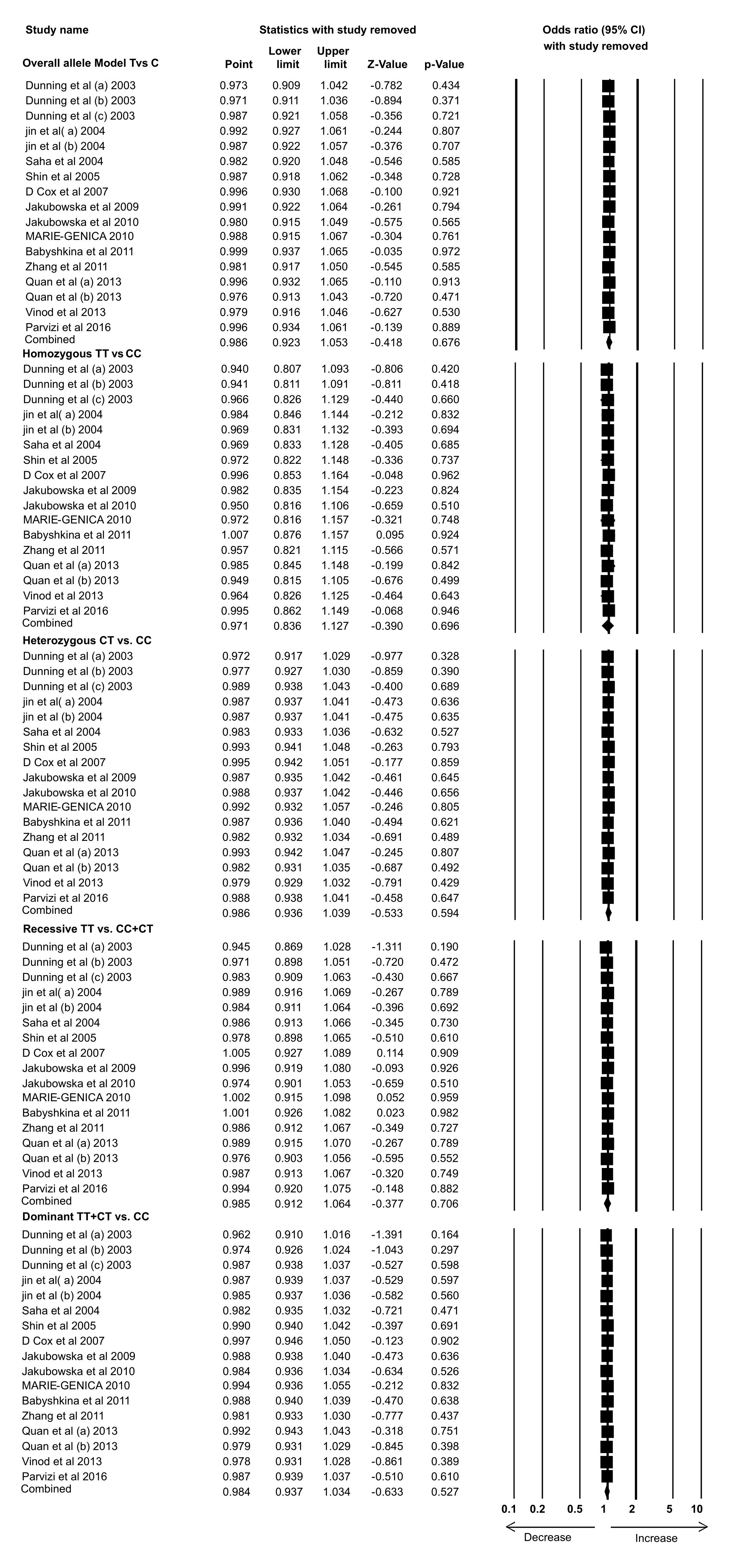

Supplement: Supplementary file 1 [file cancers-12-00471-s001.zip › Supplementary data/Supplementary Fig 6.tiff]

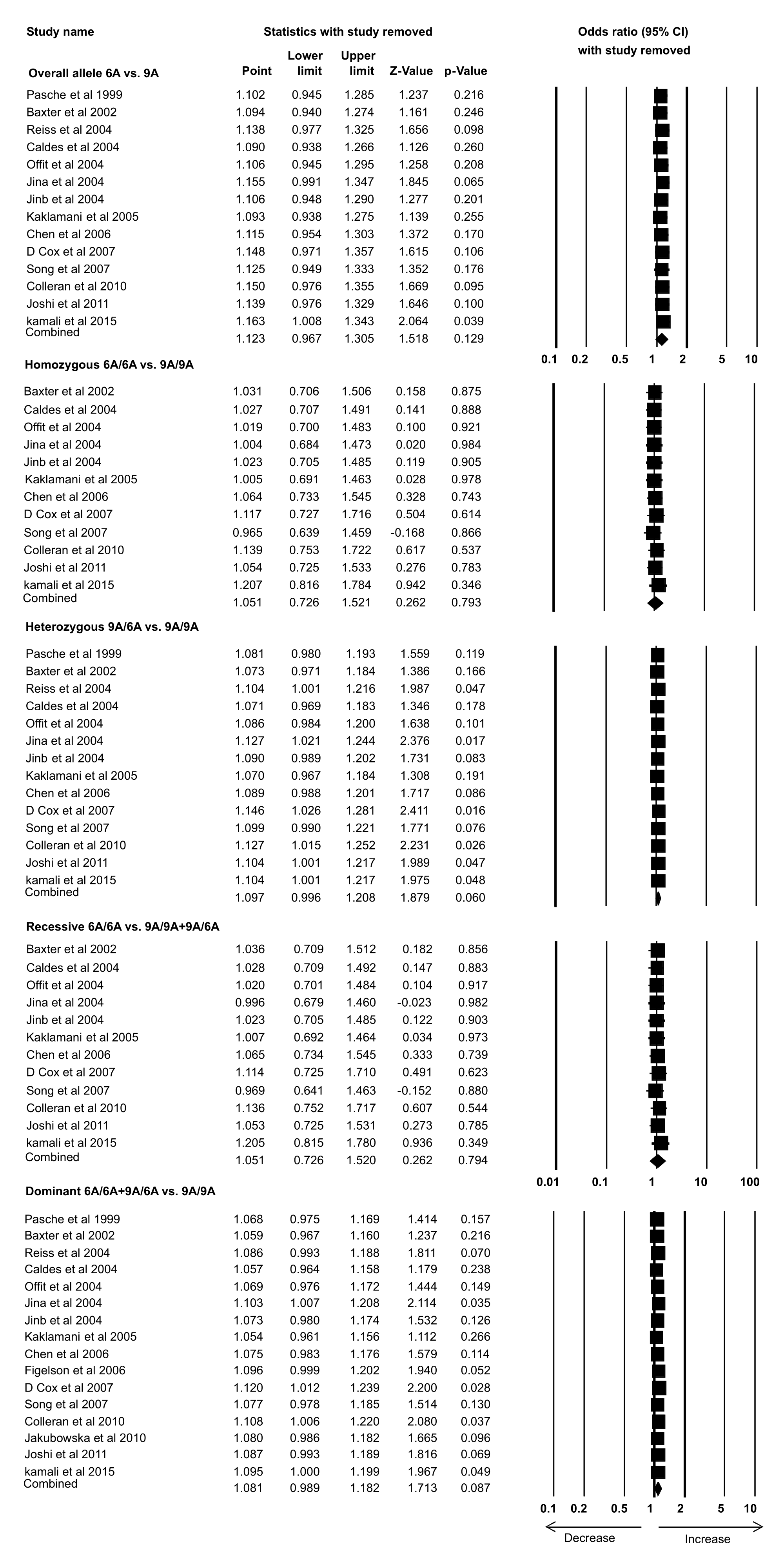

Supplement: Supplementary file 1 [file cancers-12-00471-s001.zip › Supplementary data/Supplementary Fig 7.tiff]

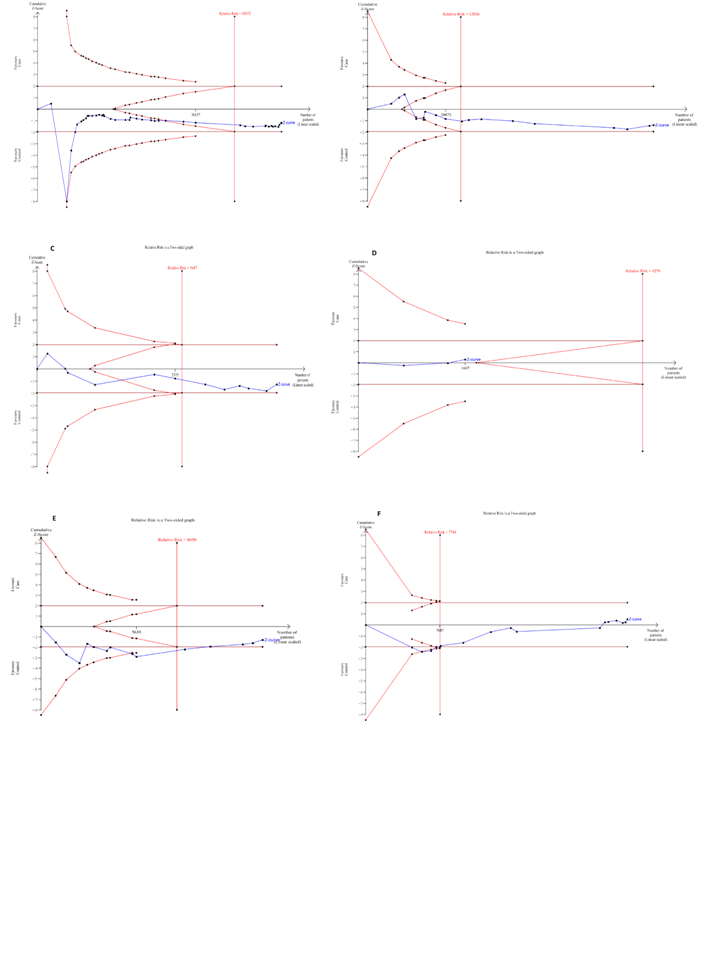

Supplement: Supplementary file 1 [file cancers-12-00471-s001.zip › Supplementary data/Supplementary Fig 8.tif]
